# Hepatomegaly Associated with Non-Obstructive Sinusoidal Dilation in Experimental Visceral Leishmaniasis

**DOI:** 10.3390/pathogens10111356

**Published:** 2021-10-20

**Authors:** Kota Maeda, Sonya Sadoughi, Ayako Morimoto, Kazuyuki Uchida, James K. Chambers, Chizu Sanjoba, Junya Yamagishi, Yasuyuki Goto

**Affiliations:** 1 Laboratory of Molecular Immunology, Department of Animal Resource Sciences, Graduate School of Agricultural and Life Sciences, The University of Tokyo, Tokyo 113-8657, Japan; poinkare@g.ecc.u-tokyo.ac.jp (K.M.); morimoto.ayako@mail.u-tokyo.ac.jp (A.M.); asanjoba@g.ecc.u-tokyo.ac.jp (C.S.); 2 Department of Veterinary Medicine, University of Cambridge, Cambridge CB3 0ES, UK; ss2482@cam.ac.uk; 3 Laboratory of Veterinary Pathology, Department of Veterinary Medical Sciences, Graduate School of Agricultural and Life Sciences, The University of Tokyo, Tokyo 113-8657, Japan; auchidak@g.ecc.u-tokyo.ac.jp (K.U.); achamber@g.ecc.u-tokyo.ac.jp (J.K.C.); 4 Division of Collaboration and Education, International Institute for Zoonosis Control, Hokkaido University, Sapporo 001-0020, Japan; junya@czc.hokudai.ac.jp; 5 International Collaboration Unit, International Institute for Zoonosis Control, Hokkaido University, Sapporo 001-0020, Japan

**Keywords:** visceral leishmaniasis, hepatomegaly, sinusoid, edema

## Abstract

Visceral leishmaniasis (VL) is the most severe form of leishmaniasis caused by protozoan parasites of the genus *Leishmania*. Hepatomegaly is one of the most frequent clinical manifestations of VL, whereas immunopathology of the symptom has not been well investigated. Using our chronic model of experimental VL, we examined the influence of *Leishmania donovani* infection on the liver by clinical, histological, and biochemical analyses. The infected mice showed increased liver weight 24 weeks post-infection. Although an increase in serum ALT and inflammatory cell accumulation were observed in the livers of infected mice, no apparent parenchymal necrosis or fibrosis was observed. Tissue water content analyses demonstrated that increased liver weight was predominantly due to an increase in water weight. Together with the finding of hepatic sinusoidal dilation, these results suggested that edema associated with sinusoidal dilation causes hepatomegaly in *L. donovani* infection. Immunostaining of platelets and erythrocytes showed no thrombus formation or damage to the sinusoidal endothelium in the liver of infected mice. Taken together, these results suggest that hepatomegaly during experimental VL is caused by non-obstructive sinusoidal dilation.

## 1. Introduction

Leishmaniasis is one of the neglected tropical diseases and is estimated to affect 0.7–1 million people annually [1]. It typically presents in either the visceral, cutaneous, or mucocutaneous forms. Of these, the visceral form, also known as visceral leishmaniasis (VL) or kala-azar, is known to show the most severe clinical disease. In 2019, more than 90% of new cases reported to the WHO occurred in ten countries: Brazil, Ethiopia, Eritrea, India, Iraq, Kenya, Nepal, Somalia, South Sudan, and Sudan [1]. Symptoms of VL include enlargement of the spleen and liver, pancytopenia, fever, and loss of body weight, which endanger the patients’ lives [1]. Among all the *Leishmania* species, *Leishmania donovani* and *Leishmania infantum* are well known to infect internal organs and cause VL.

Enlargement of the liver (hepatomegaly) is a common symptom in VL patients; 30–100% of VL patients with parasitological confirmation present with liver enlargement [2,3,4,5,6,7,8,9,10,11]. In addition, biochemical markers of hepatocellular damage, AST and ALT, are found to be elevated in the blood of these patients [2,9,10,11,12]. Moreover, it has been reported that liver failure may be a direct cause of death in VL patients [13]. Because hepatomegaly is the most common finding in the liver of VL patients, elucidation of the mechanism by which this occurs will be a good start to understanding various influences that the parasites have on the liver, potentially including portal hypertension and hepatocyte necrosis. However, there are currently a limited number of papers in the literature describing histological changes of the liver in human patients in detail, reporting hyperplasia of Kupffer cells and hepatocytes or fatty change of hepatocytes [14,15].

Although experimental models of VL are useful to study protective responses against *Leishmania* infection, they have received less attention as a tool for studying the immunopathology of VL. Despite the fact that hepatic abnormalities are characteristics of chronic disease in human VL patients [13], much research has focused not on experimental reproduction of such manifestations of chronic VL, but rather on granuloma-mediated protective immunity in the liver during the first four to eight weeks of experimental infection [16,17,18]. The latter models often use a combination of C57BL/6 mice as hosts and *Leishmania* amastigotes as infecting pathogens, and result in self-limiting liver infection with a peak parasite burden at approximately four weeks post-infection [19,20]. Hamster models of experimental VL generally show more severe pathology than mouse models, making hamsters more suitable for pathological research on VL. However, hamsters do not necessarily develop hepatomegaly during experimental VL [21]. In contrast, we have developed a mouse model of VL showing sustained parasite infection in the liver associated with non-healing hepatomegaly for up to six months [22]. Our mouse model showing prolonged hepatomegaly may be useful for studying the immunopathology of not acute, but rather chronic and sustained hepatomegaly found in VL patients.

In general, hepatomegaly is caused by various mechanisms, including congestion, portal hypertension, and hepatocyte hypertrophy [23]. These are secondary to changes in the liver on the organ, tissue or cellular levels, though yet to be determined in the case of VL. Therefore, we examined the structural and functional changes in the liver to better understand the pathogenesis of hepatomegaly in our experimental model of VL.

## 2. Results

### 2.1. Hepatic Changes Induced by Long-Term Infection with L. donovani in Mice

**Figure 1:**
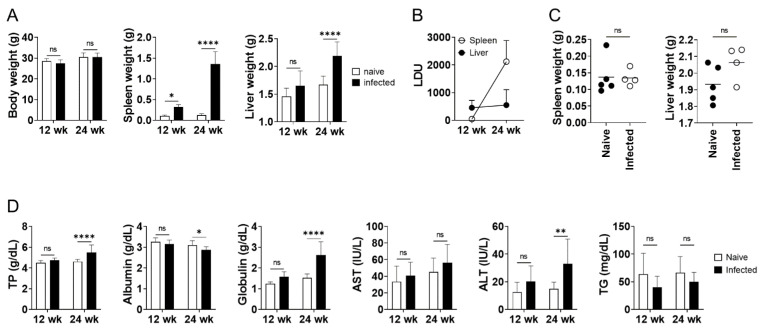
Hepatomegaly in a mouse model of chronic VL. (**A**) Body weights, spleen weights, and liver weights of naive BALB/cA mice (n = 5) and *L. donovani*-infected BALB/cA mice (n = 5 or 6) at 12 and 24 weeks post-infection. (**B**) Spleen and liver LDU of *L. donovani*-infected BALB/cA mice at 12 and 24 weeks post-infection. (**C**) Spleen weights and liver weights of naive nude mice (n = 5) and *L. donovani*-infected nude mice (n = 4) at 24 weeks post-infection. (**D**) Serum levels of indicated molecules in naive BALB/cA mice (n = 5 or 10) and *L. donovani*-infected BALB/cA mice (n = 5 or 10) at 12 and 24 weeks post-infection. Bars represent the means of individual groups. * *p* < 0.05; ** *p* < 0.01; **** *p* < 0.0001; ns, not significant by unpaired *t* test (**C**) or two-way ANOVA with Sidak’s multiple comparisons test (**A,D**). This is a representative of three independent experiments with similar results.

As previously reported [22], six months infection with *L. donovani* in BALB/cA mice induced a significant increase in the weight of the spleen and liver at 24 weeks post-infection, whereas no apparent change in body weight was induced by the infection (Figure 1A). The mean liver weight of the infected mice at 24 weeks post-infection was 31% higher than that of uninfected mice (Figure 1A). Hepatomegaly was not apparent in *L. donovani*-infected BALB/cA mice at 12 weeks post-infection, while the mean spleen weight of the infected mice was around three times as high as that of naive mice at this time point (Figure 1A). Parasite burdens in the spleen of infected mice showed a significant increase from 12 weeks to 24 weeks post-infection, whereas liver parasite burdens were equivalent between the two time points (Figure 1B). Hepatosplenomegaly caused by *L. donovani* infection was suppressed in nude mice even at 24 weeks post-infection (Figure 1C). Liver LDU of nude mice at 24 weeks post-infection (825 ± 623) was equivalent to BALB/cA mice at the same time point of infection. Serum biochemical tests demonstrated a marked increase in total protein in 24-week-infected BALB/cA mice, mainly due to an increase in globulins, but not albumin; serum albumin levels were rather lower in the infected mice (Figure 1D). Serum from the infected mice also contained mildly elevated levels of liver enzyme ALT (Figure 1D). The concentration of serum triglycerides was not significantly different between the uninfected and infected groups at 24 weeks of infection (Figure 1D). Any of the changes found in the infected mice at 24 weeks post-infection were not evident at 12 weeks post-infection (Figure 1D). Histological analyses of the liver of the 24-week-infected BALB/cA mice showed vasculitis in the portal veins and granuloma formation (Figure S2). In contrast, no apparent parenchymal necrosis or fibrosis, and no apparent changes in the number of KI-67-positive hepatocytes was observed in the infected liver (Figure S2).

**Figure 2:**
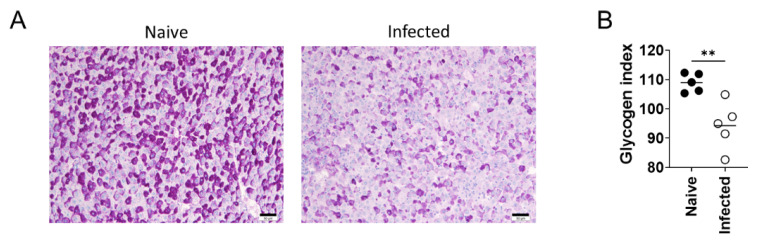

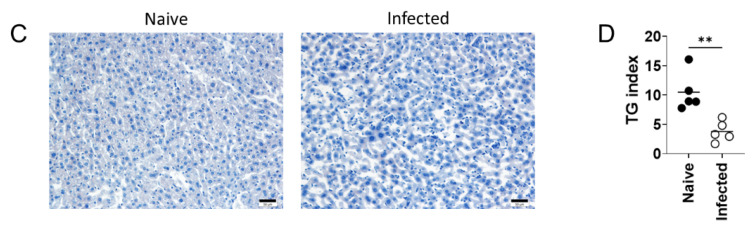
Decreased glycogen storage in the liver during experimental VL. (**A**) PAS-stained liver tissues from naive and *L. donovani*-infected BALB/cA mice at 24 weeks post-infection (n = 5 for each group). Scales, 50 μm. (**B**) Glycogen index, quantification of PAS staining intensity for liver tissues of naive and infected mice. (**C**) Oil Red O-stained liver tissues from naive and *L. donovani*-infected BALB/cA mice at 24 weeks post-infection (n = 5 for each group). Scales, 50 μm. (**D**) TG index, quantification of Oil Red O staining intensity for liver tissues of naive and infected mice. Bars represent the means of individual groups. ** *p* < 0.01 by unpaired *t* test. This is representative of three independent experiments with similar results.

The significant increase in weight, as well as the change in serum ALT levels, indicated structural and functional changes of the liver of the 24-week-infected BALB/cA mice. To investigate metabolic changes in the liver of the infected mice, PAS staining and Oil red-O staining were performed on tissue sections to visualize glycogen and triglyceride storage, respectively. PAS staining in naive mice showed that the cytoplasm of many hepatocytes was filled with granular structures that stained reddish purple, whereas there were fewer stained cells in the liver of the infected mice, and the bodies stained in these cells were only sparsely present in the cytoplasm (Figure 2A). Quantification of the PAS staining intensity showed that the signal was significantly weaker in the liver of the infected mice than that of naive mice (Figure 2B). Although TG index showed a statistical difference between naive and infected mouse livers, the values were very low in both groups, and Oil red-O staining microscopically showed no fat droplets in either naive or infected mouse livers (Figure 2C,D).

### 2.2. Hepatic Edema Due to Sinusoidal Dilation in L. donovani Infection

**Figure 3:**
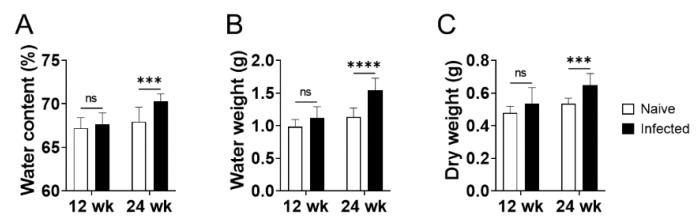
Increased water content in the liver of *L. donovani*-infected BALB/cA mice. Pieces of liver from naive and *L. donovani*-infected BALB/cA mice (n = 5 for each group) at 12 and 24 weeks post-infection were weighed before and after a drying process. Water content (**A**), water weight (**B**), and dry weight (**C**) of the whole liver were then calculated. Bars represent the means of individual groups. *** *p* < 0.001; **** *p* < 0.0001 by two-way ANOVA with Sidak’s multiple comparisons test.

To address the mechanisms for increased liver weight in BALB/cA mice infected with *L. donovani* at 24 weeks post-infection, water content of the liver from the uninfected and infected mice was examined. The water content of the infected liver was 70% and was significantly higher than that of naive mice (68%). Increased water weight (0.40 g), rather than dry weight (0.12 g), predominantly accounted for the increased whole liver weight (0.52 g) induced by *L. donovani* infection (Figure 1C and Figure 3). On the other hand, changes in water content, water weight, or dry weight of the liver were not apparent at 12 weeks post-infection (Figure 3).

**Figure 4:**
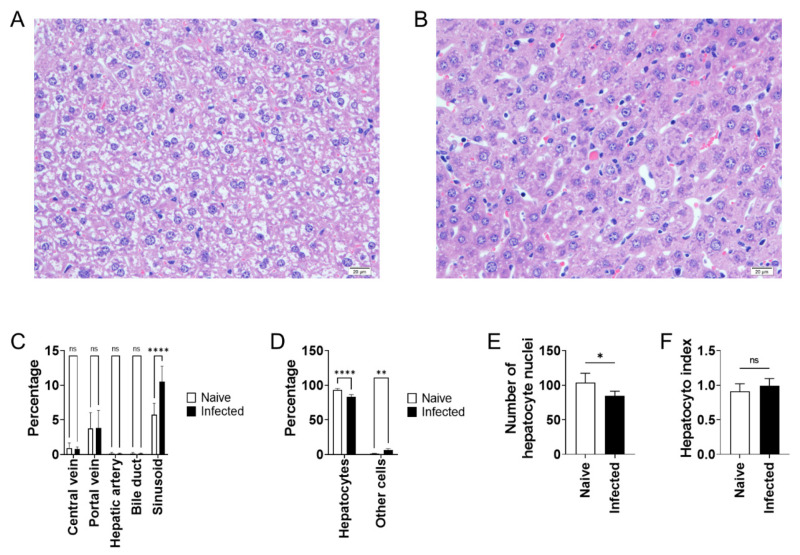
Sinusoidal dilation in BALB/cA mice with *L. donovani* infection. (**A**,**B**) Representative images of H&E-stained liver sections of naive (**A**) and *L. donovani*-infected BALB/cA mice at 24 weeks post-infection (**B**). Sinusoidal dilation in the infected liver was prominent. Scales, 20 μm. (**C**) Area occupancy by central veins, portal veins, hepatic arteries, bile ducts, and sinusoids in the liver of naive (n = 5) and *L. donovani*-infected BALB/cA mice (n = 6) at 24 weeks post-infection was histologically analyzed. (**D**) Area occupancy by hepatocytes and other cells in the liver of naive and *L. donovani*-infected BALB/cA mice at 24 weeks post-infection was histologically analyzed. Bars represent the means and SDs of individual groups. ** *p* < 0.01; **** *p* < 0.0001; ns, not significant by two-way ANOVA with Sidak’s multiple comparisons test. (**E**) The average numbers of hepatocytes and other cells per field in the liver of naive and *L. donovani*-infected BALB/cA mice were histologically analyzed. (**F**) Hepatocyte index, which is an indicator of hepatocyte cell size, is shown. Bars represent mean ± SD of individual groups. * *p* < 0.05; ns, not significant by unpaired *t* test.

Histological observations did not reveal any apparent hepatocyte hypertrophy in the 24-week-infected liver. Next, to seek out other mechanisms to explain the water increase in *L. donovani* infection, blood vessel dilation was looked for histologically. Image analyses of area occupancy by arteries, central veins, portal veins, and bile ducts demonstrated no dilation in any of these in *L. donovani* infection (Figure 4A). On the other hand, area occupancy by sinusoids was high in the liver of the infected mice at 24 weeks post-infection (Figure 4A). Conversely, the area occupied by hepatocytes was lower in the infected liver, and this decline was also accompanied by a decrease in the number of hepatocyte nuclei (Figure 4B, C). The hepatocyte index, an indicator of the average size of hepatocytes, was comparable between the uninfected and infected mice (Figure 4D), indicating that increased sinusoidal space is not due to shrinkage of hepatocytes.

Further histological analyses did not demonstrate any obvious venous obstruction. Immunohistochemical staining of erythrocytes and platelets did not show increased aggregation of either within sinusoids of the infected liver (Figure S3), suggesting that thrombus formation and subsequent sinusoidal obstruction are unlikely. Platelets also did not extravasate into the space of Disse in the infected liver, suggesting minimal damage to the sinusoidal endothelia.

## 3. Discussion

Hepatomegaly is one of the major clinical manifestations of VL. Using a mouse model of *L. donovani* infection with long-term persistence of the parasites in the liver, we demonstrated that hepatomegaly during chronic VL is caused by edema accompanied with sinusoidal dilation. Increased liver weight during *L. donovani* infection in BALB/cA mice was significant at 24 weeks of infection but not at 12 weeks (Figure 1). On the other hand, liver parasite burdens were equally high at 12 and 24 weeks post-infection, suggesting that hepatomegaly is not just a reflection of parasite burden in the liver. The water content of the liver increased from 68 to 70% in 24-week-infected mice and this increase in water weight accounted for the majority of the increase in whole liver weight (Figure 3). While accelerated proliferation or hypertrophy of hepatocytes was not evident in the infected liver when analyzed (Figure 4 and Figure S2), sinusoidal dilation was remarkable in the infected tissue, suggesting that the mass of extracellular fluid, rather than cellular mass, contributed to the increase in liver weight in *L. donovani* infection. It is intriguing that vascular dilation was unique to sinusoids: no obvious dilation of central or portal veins was found and no apparent emboli were detected in any of the sinusoids, central veins, or portal veins of the liver of infected mice (Figure 4). Hepatocyte shrinkage was also not detected (Figure 4), and serum levels of AST and ALT were unchanged or only mildly elevated in infected mice (Figure 1), suggesting that the dilation of sinusoids does not result from damage to hepatocytes and may be induced by mechanisms directly targeted to the hepatic sinusoids. Sinusoidal dilation, as found in our experimental model, has also been reported by another group in *L. donovani*-infected C57BL/6 mice [20], suggesting that this pathological change is not restricted by the genetic background of mice.

Lima et al. have previously reported histological alterations in the liver of dogs naturally infected with *L. infantum* [24], and many of the alterations found in dogs, including portal inflammation and granuloma formation, were shared with the findings of our study. Portal inflammation has also been reported in human VL with a high prevalence [14]. Low frequencies of sinusoidal congestion, hepatocyte steatosis, and hepatocyte necrosis were also found in the dogs [24], with similar patterns to the mice in this study. In humans, Datta et al. reported sinusoidal dilation in the liver of a VL patient [25]. Prasad et al. also reported a high prevalence of sinusoidal dilation in liver of VL patients [26]. However, such studies on liver histology in human VL are very limited, and it is difficult to estimate the true prevalence of hepatic sinusoidal dilation during the disease. In dogs, sinusoidal dilation was found only in ~20% of VL cases [24]. The low prevalence may suggest that sinusoidal dilation is a characteristic of not mild but severe VL. Hepatomegaly itself may also be an indicator of more advanced VL as its prevalence in human cases is generally lower than that of anemia or splenomegaly [2,3,6,7,27]. In our study, hepatomegaly accompanied by an increase in water weight became evident at 24 weeks post infection in mice, but not 12 weeks (Figure 1 and Figure 3). At 12 weeks post-infection, the spleen weight of infected mice was already three times as high as that of naive mice (Figure 1), demonstrating a delayed onset of hepatomegaly relative to splenomegaly.

It was revealed that hepatomegaly and splenomegaly, the two organomegalies evident during VL, are immune-mediated as these disease manifestations were not seen in nude mice with *L. donovani* infection (Figure 1). In fact, El Hag et al. also indicated immunological etiology of liver pathology in VL based on their clinical findings [14]. Hepatomegaly appears to be less common in HIV-coinfected VL patients than HIV-negative VL patients [28], and Ramos et al. showed that the incidence of hepatomegaly in VL was even lower in immunosuppressed individuals than those with HIV coinfection [29]. However, mechanisms for the enlargement of spleen and liver during experimental VL may be quite different and these organomegalies may occur as independent events. Firstly, splenomegaly during experimental VL is accompanied by an increase in cell numbers [30], whilst hepatomegaly is not - as demonstrated in the present study. Secondly, our previous studies using genetically modified mice demonstrated that deficiency in host molecules BAFF and MRP14 leads to attenuation of splenomegaly, but not hepatomegaly, in *L. donovani* infection [30,31]. Although it is known that dysfunction or damage in the liver or the spleen often affects the other, the influence of *L. donovani* infection on these organs does not appear very closely connected. Carrion et al. demonstrated that the liver weight in *L. infantum*-infected BALB/cA mice continued to increase at day 56 of infection, whilst liver parasite burden peaked at day 14 [32]. Along with our data in nude mice, it should be noted that hepatomegaly in experimental VL is not necessarily associated with an increase in parasite burden.

There are several models of hepatic sinusoidal dilation. Marzano et al. proposed possible causes as obstruction of the sinusoids or central veins, including thrombosis due to damage to the sinusoidal endothelium, and non-obstructive mechanisms including hepatocyte shrinkage, increased inflow of blood, and an increase in soluble factors, which induce sinusoidal dilation [33]. One of the best known is sinusoidal obstruction; sinusoidal obstruction syndrome is caused by swelling and detachment of the sinusoidal endothelium, leading to hypercoagulability and obstruction of the sinusoids [33]. Aberrant production of immunoglobulins during experimental VL [30] implies that the coagulation system is hyper-activated in the infected mice and that the endothelium is damaged by inflammation. Contrary to this, however, there was no apparent thrombosis (Figure S3), nor was there any evidence of platelet extravasation and aggregation in the space of Disse (Figure S3), which would otherwise be an indicator of damage to the sinusoidal endothelium. Therefore, it is necessary to consider non-obstructive causes of sinusoidal dilation. For example, nitric oxide induces dilation of hepatic sinusoids in various conditions [34]. During experimental VL, *nos2* expression was highly up-regulated in the infected liver (data not shown). In addition to nitric oxide, carbon monoxide is also involved in vasodilation in the liver [35,36]. The infected liver also had up-regulated expression of *hmox1* gene (data not shown), which encodes the enzyme involved in production of carbon monoxide. Future studies on nitric oxide and carbon monoxide, as well as other soluble factors, may help to elucidate the detailed mechanisms of sinusoidal dilation leading to hepatomegaly during experimental VL.

The fact that hepatomegaly is a major symptom of VL does not mean that this enlargement is the only change in the liver during VL. To address other hepatic changes that may occur in *L. donovani* infection, serum biochemical analyses as well as histological analyses were performed. One finding was the reduction of serum albumin in the infected mice (Figure 1). Low levels of albumin are also reported in human VL patients [37]. As production of albumin mainly occurs in the liver, the lower levels seen in infected mice and human VL patients indicate functional dysregulation of the liver by *Leishmania* infection. Additionally, there was a significant reduction in glycogen storage in the infected mice, suggestive of altered metabolism during experimental VL. Since IL-6 affects hepatocytes and causes insulin resistance [38], it is possible that infection-induced inflammation results in insulin resistance. However, steatosis was not observed in the infected liver, suggesting that insulin resistance is unlikely (Figure 2). Increased serum ALT in infected mice indicates a certain level of damage to hepatocytes by *L. donovani* infection (Figure 1). Nonetheless, histological analyses and TUNEL staining (data not shown) did not reveal enhanced necrosis or apoptosis of hepatocytes by the infection. This suggests that it is not a change in cell number, but rather, altered cell signaling in hepatocytes that is likely to account for the observed metabolic changes in the infected liver.

In summary, we demonstrated that hepatomegaly in experimental chronic VL is due to edema associated with sinusoidal dilation. The dilation is not caused by either thrombosis or hepatocyte shrinkage, but may instead be caused by mechanisms unique to hepatic sinusoids. Besides, *L. donovani* infection causes other functional changes in the liver, including storage of glycogen. Although there is no guarantee that our animal model surely reproduces liver pathology found in clinical VL cases, the experimental model provides some similarity to findings in human and dog VL. Therefore, it should be somewhat useful for understanding pathogenesis of hepatomegaly in chronic VL, because analyses of hepatomegaly kinetics in clinical VL cases requiring multiple biopsy collections are ethically difficult, as any interventions for understanding the pathogenesis are unacceptable if they do not provide treatment. We believe that weakness of animal models of VL will be overcome by further collection of data in the experimental models coupled with careful comparisons with findings in clinical VL.

## 4. Materials and Methods

### 4.1. Mice and Parasites

Female BALB/cA mice and BALB/cA-nu/nu (nude) mice were purchased from Japan Clea, Tokyo, Japan. All mice were maintained under specific pathogen-free conditions. The mice were used for experiments at the age of 6–8 weeks. *Leishmania donovani* promastigotes (MHOM/NP/03/D10; a gift from the National BioResource Project at Nagasaki University [39]) were cultured in medium 199 (Thermo Fisher Scientific #12350-039, Waltham, MA, USA) supplemented with 10% heat-inactivated fetal bovine serum (Thermo Fisher) and 100 units/mL of penicillin and 100 µg/mL of streptomycin (Thermo Fisher) at 25 °C.

### 4.2. Experimental Infection, Hematological Analyses, and Autopsy

*L. donovani* promastigotes (passage number = 2) in late log or stationary phase were washed with phosphate-buffered saline (PBS: Nissui Pharm, Tokyo, Japan) by centrifugation at 1600× *g* for 10 min and were resuspended with PBS at the concentration of 1 × 10^8^ cells/mL. Mice were infected with 1× 10^7^ *L. donovani* promastigotes by intravenous injection into the tail vein. After 12 or 24 weeks, cardiac puncture was performed on those mice under isoflurane anesthesia to collect the whole blood at the time of euthanasia, and the spleen and liver were then harvested. Impression smears of the spleen and liver were fixed for 5 min in methanol and stained for 25 min with 5% Giemsa solution (Merck KGaA, Parmstadt, Germany). Amastigotes were counted by microscopic observation of the stained smear at 1000× magnification, and Leishman-Donovan Units (LDU) were enumerated as the number of amastigotes per 1000 host nuclei times the tissue weight in grams as performed in a previous study [40].

### 4.3. Periodic Acid Schiff (PAS) Staining

The tissues collected at the time of sacrifice were fixed with Carnoy’s solution composed of 60% methanol, 30% chloroform, and 10% acetic acid for 48 h and embedded in paraffin. Four-micrometer-thick sections were prepared from the paraffin-embedded tissues. The sections were dewaxed and treated with periodic acid for 5 min. Next, the sections were covered with Schiff’s reagent (Muto Pure Chemicals Co., Ltd. Tokyo, Japan) for 20 min and with sulfite water for 6 min for color development. Then, they were stained with Mayer’s hematoxylin solution (WAKO) for 90 s and rinsed in running tap water for 5 min. For the quantification of glycogen, images were split into three colors using a Fiji plugin “Colour Deconvolution” (set vector at “H PAS”), purple signals were inverted to monochrome, and average color intensity was quantified.

### 4.4. Oil Red-O Staining

The tissues collected at the time of sacrifice were fixed with 10% buffered formalin (Sumitani Shoten Co., Ltd., Osaka, Japan). The fixed tissues were sent to Biopathology Institute Co., Ltd. (Oita, Japan) to make frozen sections and stain neutral fat with Oil red-O. For the quantification of triglyceride (TG) images were split to three colors using a plugin “Colour Deconvolution” (set vector at “RGB”), red signals were inverted to monochrome, and average color intensity was quantified.

### 4.5. Histological Analyses

The tissues collected at the time of sacrifice were fixed with 10% buffered formalin and then embedded in paraffin. Four-micrometer-thick liver sections were dewaxed and stained with hematoxylin and eosin (H&E) for histopathological and stereological analyses. Digital images of hepatic tissue were obtained using a light microscope (Olympus BX-53, Tokyo, Japan) connected to a digital camera (Olympus DP73, Tokyo, Japan). Eight photomicrographs from H&E-stained sections (10×, 40× objective) were randomly obtained for each mouse. For the stereological analysis, a test system of 192 or 768 points was used in a standard test area [41,42]. In sections stained with H&E, the points were recorded in liver components (hepatocytes, sinusoidal capillaries, blood vessels, and others). Area occupancy by individual components was calculated as the proportion of dots for the corresponding component from the total dots (Supplementary Figure S1). In addition, the number of nuclei in the micrograph was counted. Hepatocyte index, an indicator of the average size of hepatocytes, was calculated as below.

(1) $$\mathrm{Hepatocyte}\;\mathrm{index}\;=\frac{\mathrm{Hepatocyte}\;\mathrm{index}\;\left(\%\right)}{\mathrm{Number}\;\mathrm{of}\;\mathrm{hepatocyte}\;\mathrm{nuclei}}$$

### 4.6. Immunohistochemical Analyses

Immunohistochemical staining was performed to characterize the nuclei of proliferating cells. The tissues collected at the time of sacrifice were fixed with Carnoy’s solution for 48 h and embedded in paraffin. Paraffin-embedded tissues were dewaxed and boiled in 10 mM sodium citrate buffer (pH 6.0) for 20 min, followed by washing with tap water. Endogenous peroxidase was inactivated with 0.3% H_2_O_2_ in methanol for 30 min. After blocking with Block Ace (DS Pharm., Osaka, Japan), 2.5 μg/mL solution of anti-mouse KI-67 antibody (Thermo Fisher #14-5698-80), 6 μg/mL solution of anti-mouse CD42b antibody (Abcam #ab183345) and 0.5 μg/mL solution of anti-mouse Ter119 antibody (BioLegend #116211) was applied to the sections of liver, and the sections were incubated for 1 hour at room temperature and washed with PBS. Next, Histofine^®^ Simple Stain Mouse MAX PO (Rat) (Nichirei, Tokyo, Japan), or Histofine^®^ SAB PO(R) kit second antibody (Nichirei, Tokyo, Japan) followed by HRP-conjugated streptavidin (Nichirei, Tokyo, Japan) was applied, and the sections were incubated for 1 h at room temperature and washed with PBS. After incubating with 3,3’-diaminobenzidine (DAB) substrate (Nichirei) to yield an enzymatic color change, the sections were counterstained with Mayer’s hematoxylin solution for 60 s and rinsed with tap water for 40 min. The pictures of each section were split to blue, blown, and green using a Fiji plugin “Colour Deconvolution” (set vector at “HDAB”), and the number of KI-67 positive and negative nuclei was counted.

### 4.7. Measurement of Liver Water Content

The cut tissue pieces (~100 mg of liver for each mouse) were placed at the bottom of a 1.5 mL centrifuge tube (BIOBIK #CF-0150) and dried at 90 °C for 24 h using a CTU-mini (TAITEC, Saitama, Japan). Of note, the dry weight did not change by further drying after 24 h. The dry weight of the whole liver was calculated as the product of whole liver wet weight and the dry: wet weight ratio of the cut piece. The water content of each sample was calculated as below.

(2) $$\mathrm{Water}\;\mathrm{content}\;\left(\%\right)=\frac{\mathrm{Wet}\;\mathrm{weight}\;–\;\mathrm{Dry}\;\mathrm{weight}}{\mathrm{Wet}\;\mathrm{weight}}\times 100$$

### 4.8. Serum Biochemical Tests

Serum was separated from the collected blood and analyzed for biochemical tests by Oriental Yeast Co., Ltd. (Tokyo, Japan). The following methods were used for the individual tests; total protein by biuret method, albumin by BCG method, globulin calculated as (total protein-albumin), AST by JSCC transferable method, ALT by JSCC transferable method, and TG by the GPO-HMMPS method.

### 4.9. Statistical Analysis

Statistical analysis was performed using GraphPad Prism 9 software package (GraphPad Software Inc., San Diego, CA, USA). Results are presented as mean ± standard deviation (SD). Either unpaired *t* test or two-way ANOVA coupled with Sidak’s multiple comparisons test was used for comparison of two independent groups, i.e. naive and *L. donovani*-infected mice. *p* values less than 0.05 were considered statistically significant.

## Data Availability

Data presented in this study will be available from the corresponding author upon request.

## Supplementary Materials

The following are available online at https://www.mdpi.com/article/10.3390/pathogens10111356/s1, Figure S1: Illustration of area occupancy analyses., Figure S2: Histological and immunohistochemical analyses on the liver of *L. donovani*-infected mice., Figure S3: No apparent obstruction of the sinusoids in the liver of *L. donovani*-infected mice.
