# Supplementary material for: Hepatomegaly Associated with Non-Obstructive Sinusoidal Dilation in Experimental Visceral Leishmaniasis
Source: Pathogens. 2021 Oct 20;10(11):1356. doi: 10.3390/pathogens10111356 (PMC8625948; doi:10.3390/pathogens10111356)
Supplement: Supplementary file 1 [file pathogens-10-01356-s001.zip › pathogens-1364844-supplementary.pdf]

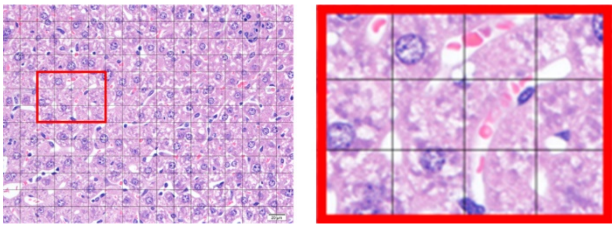

Figure S1. Illustration of area occupancy analyses. Photomicrographs from H&E (10×, 40× objective) were randomly obtained for each group. For the quantitative histological analysis, a test system of 192 or 768 points was used in a standard test area. Left, an example of the 192-point system; right, a magnified image of the boxed area of the left. In sections stained with H&E, the points were recorded in liver components (hepatocytes, sinusoidal capillaries, blood vessels, and others). Area occupancy by individual components was calculated as the proportion of dots for the corresponding components from the total dots.

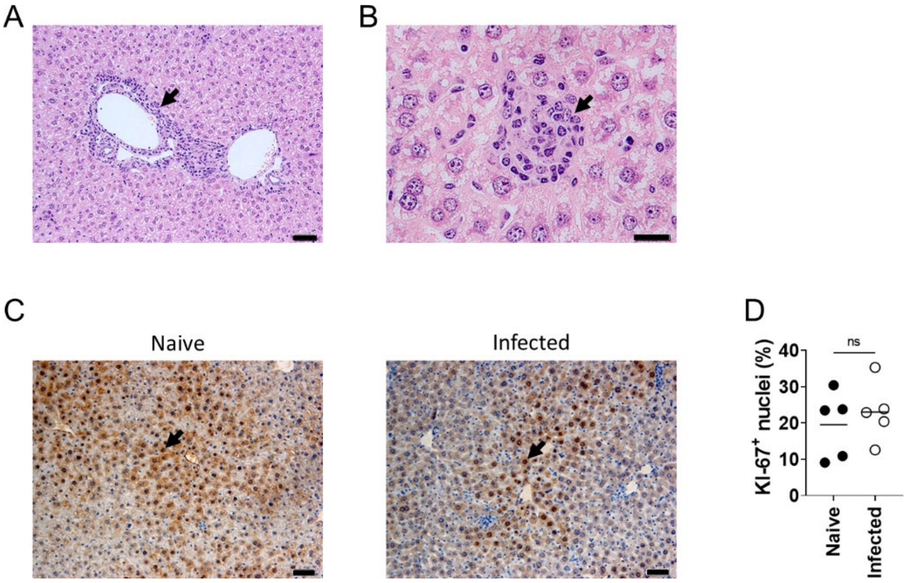

Figure S2. Histological and immunohistochemical analyses of the liver of *L. donovani*-infected mice. (A, B) Images of H&E-stained sections on liver tissue from *L. donovani*-infected mice at 24 weeks post-infection are shown. (A) Vasculitis of the portal veins. Scale, 50 μm. (B) Granuloma formation. Scale, 20 μm. (C) Immunohistochemical staining for KI-67 in the liver of naïve and *L. donovani*-infected mice at 24 weeks post-infection is shown. Scales, 50 μm. (D) Percentages of KI-67-positive nuclei in the liver of naïve and *L. donovani*-infected mice.

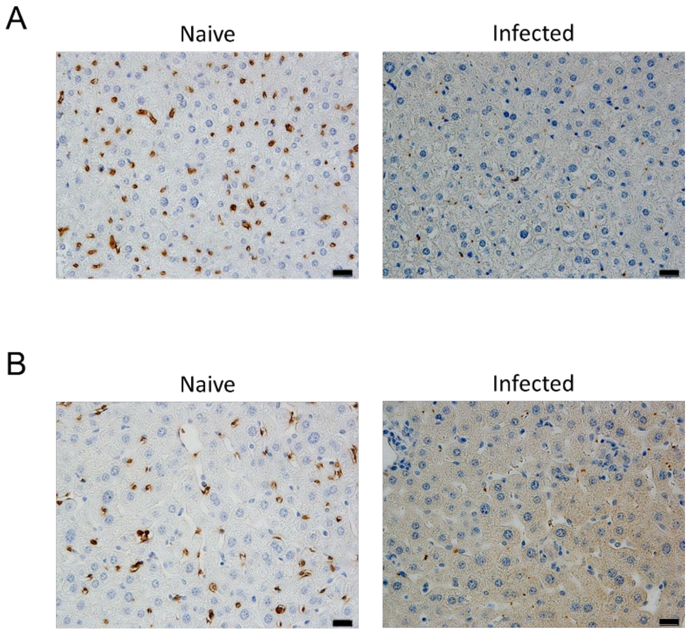

Figure S3. No apparent obstruction of the sinusoids in the liver of *L. donovani*-infected mice. Immunohistochemical staining of Ter119 for erythrocytes (A) and CD42b for platelets (B) in the liver of naïve and *L. donovani*-infected mice at 24 weeks post-infection are shown.
